# Usability and acceptability of four systematic review automation software packages: a mixed method design

**DOI:** 10.1186/s13643-019-1069-6

**Published:** 2019-06-20

**Authors:** Gina Cleo, Anna Mae Scott, Farhana Islam, Blair Julien, Elaine Beller

**Affiliations:** 1 Institute for Evidence-Based Healthcare, Bond University, Gold Coast, Australia; 2 Faculty of Health Sciences and Medicine, Bond University, Gold Coast, Australia

**Keywords:** Automation, Qualitative report, Acceptability, Usability, Software packages, Systematic Review Accelerator

## Abstract

**Aim:**

New software packages help to improve the efficiency of conducting a systematic review through automation of key steps in the systematic review. The aim of this study was to gather qualitative data on the usability and acceptability of four systematic review automation software packages (Covidence, SRA-Helper for EndNote, Rayyan and RobotAnalyst) for the citation screening step of a systematic review.

**Methods:**

We recruited three volunteer systematic reviewers and asked them to use allocated software packages during citation screening. They then completed a 12-item online questionnaire which was tailored to capture data for the software packages used.

**Findings:**

All four software packages were reported to be easy or very easy to learn and use. SRA-Helper for EndNote was most favoured by participants for screening citations and Covidence for resolving conflicts. Overall, participants reported that SRA-Helper for EndNote would be their software package of choice, primarily due to its efficiency.

**Conclusion:**

This study identified a number of considerations which systematic reviewers can use as a basis of their decision which software to use when performing the citation screening and dispute resolution steps of a systematic review.

**Electronic supplementary material:**

The online version of this article (10.1186/s13643-019-1069-6) contains supplementary material, which is available to authorized users.

## Background

Systematic reviews are the foundation of evidence-based practice. Yet, despite advancements in automation of some of the steps of systematic reviews [1, 2], conducting a systematic review remains a largely manual process that requires considerable expertise, time and financial resources. Software packages have recently become available to help to improve the efficiency of some of the steps of the systematic review process, including literature searching, de-duplicating of search results, screening citations and resolving conflicts. Each software package has its strengths and limitations. This study aimed to, firstly, assess the usability (ease of use and learnability) and acceptability (sufficient to serve the purpose for which it is intended) of the following, commonly used, systematic review automation software packages: Covidence, SRA-Helper for EndNote, Rayyan and RobotAnalyst, and secondly, to identify key advantages and disadvantages of each, as perceived by the users.

## Methods

Three volunteer systematic reviewers, who were commencing a systematic review (882 total citation records to screen), were recruited via email invitation from the study authors (GC and EB). The systematic reviewers were asked to use allocated software packages for the title/abstract screening and dispute resolution steps of the systematic review and to complete an online questionnaire which was tailored to capture data for the software package used. One participant was assigned to use and review Covidence and SRA-Helper for EndNote (BJ), and one was assigned to Rayyan and RobotAnalyst (FI). The final reviewer was assigned to use and review Covidence vs SRA-Helper for EndNote vs Rayyan vs RobotAnalyst (AMS). Each software was used to screen 220 or 221 references, for a total of 882 references screened.

Covidence (www.covidence.org) is a web-based screening and data extraction tool; it is one of the tools recommended by the Cochrane Collaboration [3]. Covidence allows authors to import and screen citations and full-text articles, resolve conflicts, extract data using customisable forms and export results in various formats.

SRA-Helper for EndNote (https://github.com/CREBP/EndNoteHelper) is a downloadable automation script which works as an add-on to EndNote; it is part of the Systematic Review Accelerator. SRA-Helper for EndNote allows users to map keyboard keys (e.g. 1, 2, 3) to folders (e.g. include, exclude, background). When the user highlights a reference (e.g. Smith 1998), and presses the key (e.g. 1), the reference automatically moves to the relevant folder (e.g. include).

Rayyan (https://rayyan.qcri.org) is a web-based application which allows multiple authors to create and collaborate on systematic reviews. Throughout the citation screening process, Rayyan offers suggestions for article inclusion based on the authors’ prior selections [4].

RobotAnalyst (www.nactem.ac.uk/robotanalyst) is a web-based application, developed to support the citation screening phase of systematic reviews. RobotAnalyst prioritises references by relevancy predictions and updates the predictive model after the author makes each screening selection.

To identify the advantages and disadvantages of each software package, we used a 12-item questionnaire which included 3 Likert-scale questions and 9 free text questions (Additional file 1: Table S1). Questions 1–8 focused on the usability and acceptability of each software package; these questions were repeated for each software package being assessed. Questions 9–11 were comparator questions, which assessed the user’s preference for one software tool over another when screening citations and resolving conflicts. We used Qualtrics (www.qualtrics.com) to disseminate the questionnaires and collect the data.

## Results

The three systematic reviewers answered all of the questions presented in the qualitative questionnaire.

We summed the participants’ quantitative responses for each software package as displayed in Table 1 (lowest possible score = 2 points; highest possible score = 10 points). All four software packages were reported to be easy or very easy to learn and use (Table 1). Covidence had the highest rating for general usability, scoring 9 out of a possible 10 points. This may be due to its ‘straightforward process and simple layout’, and it being available online, without the need to download it, ‘making it versatile/accessible when out of office’. SRA-Helper for EndNote was rated fastest for response time (10/10), as ‘it’s not dependent on internet connection’ and RobotAnalyst the slowest (5/10). When combining scores for ease of learning how to use the software, usability and response time, SRA-Helper for EndNote scored highest (28/30).

**Table 1:** Sum of quantitative responses for each software package

	Covidence	SRA-Helper for EndNote	Rayyan	RobotAnalyst
How easy was it to learn how to use…^a^	8	10	10	9
How would you rate the general usability of…^b^	9	8	8	8
How would you rate the response time of…^c^	6	10	7	5
Total (out of a possible 30 points)^d^	23	28	25	22

^a^2 = very difficult, 4 = difficult, 6 = not so difficult; 8 = easy, 10 = very easy

^b^2 = not at all user friendly, 4 = not user friendly, 6 = slightly user friendly, 8 = fairly user friendly, 10 = very user friendly

^c^2 = very slow, 4 = slow, 6 = manageable, 8 = quick, 10 = very quick

^d^The greater the number, the more favourable the response

SRA-Helper for EndNote was most favoured by participants for screening citations and Covidence for resolving conflicts (Table 2). Overall, participants reported that in conducting future systematic reviews, SRA-Helper for EndNote would be their software package of choice, primarily due to its efficiency (Table 2).

**Table 2:** Comparison between software packages

	Participant 1	Participant 2	Participant 3
	Covidence vs SRA-Helper for EndNote	Rayyan vs RobotAnalyst	Covidence vs SRA-Helper for EndNote vs Rayyan vs RobotAnalyst
Which software package did you find better for screening citations? Why did you make this selection?	SRA-Helper for EndNote	Rayyan	SRA-Helper for EndNote
	I am more proficient with computers than most and so I prefer the more technical systems	Because of the annotations I am able to make	Web-based tools are too slow and do not allow mapping of keys onto decisions (have to use the mouse throughout)
Which software package did you find better for resolving conflicts? Why did you make this selection?	Covidence	RobotAnalyst	Covidence
	The automation of Covidence really streamlines the process further	Because the software’s input helped me see things from a new perspective	SRA-Helper for EndNote is not particularly great for this—it’s a bit time consuming
If you were to conduct a screening of citations and resolving conflicts again, which of the two/four software packages are you most likely to use? Why did you make this selection?	SRA-Helper for EndNote	RobotAnalyst	SRA-Helper for EndNote
	SRA-Helper for EndNote was quicker	The continuous re-calibration about estimations in order to make accurate predictions is a useful tool. Also, I enjoy knowing how many citations I have left to screen	Because of the ability to map keyboard keys onto decisions and automatic advancing of new reference to top of screen once previous reference decided
Do you have any further comments or feedback?	Nil	Nil	Nil

The key advantages considered relevant by systematic reviewers included visibility of already screened/yet to be screened citations (e.g. in a form of a countdown or summary of progress), ability to highlight key terms for inclusion and exclusion, ability to use keyboard shortcuts, fast response time of the software (e.g. between decision to include and the reference moving off the screen and into the ‘included’ folder), ability to add notes or labels to the references, guidance of decision for subsequent references on the basis of prior decisions and ease of learning how to use the tool and intuitiveness of the layout.

The key disadvantages considered relevant by the systematic reviewers included glitches in the software (e.g. crashes), slow response time of the software, inability to see progress (references screened vs those remaining to be screened), inability to change mind once decision to include/exclude was made, requirement to download or install software, inability to highlight included/excluded terms, unreliable predictions and poor layout (e.g. decision buttons too close together). The advantages and disadvantages of using each automation software package to screen citations and resolve conflicts are summarised in Table 3.

**Table 3:** Summary of the reported advantages and disadvantages of each software package

Advantages	Disadvantages
Covidence	
- Easy to learn how to use - Straightforward process and simple layout to follow, easy to use - Does not require downloading (available online), making it versatile/accessible when out of office - Countdown of screened citations - Option to highlight key words (regarding reason for including/excluding citation) - References yet to be screened, automatically move up to the top of the page - Fairly/very user friendly	- Potential bugs/glitches in software - Slow to respond once user has made a judgement to include/exclude citation - No library of categorised articles (unable to view how many articles have been included or excluded) - User must refresh the library to update the number of citations yet to screen (not automatic) - User is unable to change their mind regarding including or excluding a previous reference as the references disappear once a decision is made
SRA-Helper for EndNote	
- Very easy to learn how to use - Offer keyboard shortcuts with the ability to map buttons onto decisions - Very fast response time (only limited by the speed of your computer) - No server delays as it is not dependent on internet connection - Ability to see libraries of ‘include’, ‘exclude’ or ‘maybe’, automatically populate - Intuitive layout - Efficient for screening titles/abstracts - Fairly user friendly	- Requires downloading and installing - Bugs/crashing - May take time to learn how to use keyboard shortcuts and to create folders/map keys onto decisions
Rayyan	
- Very easy to learn how to use - Intuitive interface - Ability to highlight included keywords in green and excluded in red - Fairly user friendly - Ability to tag notes/labels to articles - Manageable/quick response rate	- Glitches in software causing delays
RobotAnalyst	
- Easy to learn how to use and easy to use - Intuitive layout - Presents an overall summary of progress (pie graph) - Live estimations tool aids in guiding responses to subsequent citations - Entire citation highlights the relevant colour once decision is made (i.e. red for exclude) - Fairly user-friendly	- Slow/manageable response time - Lacks ability to input and highlight included/excluded terms, which would quicken screening - Predictions are not always reliable, especially when few inclusions - The decision buttons (include/exclude/unsure) are small and too close together

## Discussion

Overall, the systematic reviewers found all four of the software tools easy to learn and use. SRA-Endnote helper was strongly preferred (28/30 points), with RobotAnalyst, Covidence and Rayyan scoring lower but similarly (22, 23 and 24/30, respectively). The strong preference for SRA-Endnote Helper may be explained by its ease of learning to use and very quick response time due to it being a desktop (rather than web-based) tool.

Among the key characteristics considered relevant were display of screening progress, ability to revise decisions, ability to highlight inclusion/exclusion terms, ability to use keyboard shortcuts rather than the mouse, software response time and reliability (i.e. no bugs or crashes). Users, particularly those newer to systematic reviews, also cited the intuitiveness of the layout and ease of learning how to use the tool as important.

It is worth emphasising here that what is considered an advantage or disadvantage will vary by systematic reviewer: one may prefer a web-based tool that is a bit slower in response time but available anywhere without a download or install, whilst another may prefer a downloadable tool that is faster in response time but requires a download or install. However, whilst the individual preferences will vary, our aim here was to *identify* what those key considerations are—to help systematic reviewers (particularly those new to these tools) to make their own decisions which to use.

Although the sample of systematic reviewers included in the present assessment is small (*n* = 3), it deliberately included both novice systematic reviewers and an experienced systematic reviewer. We are therefore confident that the considerations they raised are likely to be the issues considered relevant by the larger systematic review population. This can only be formally measured with a larger sample in future research.

Ongoing and future developments in the automation of screening—including automated screening and text mining—would help to increase the efficiency and reduce human effort required [1, 2].

## Conclusion

The results of this qualitative report highlight multiple advantages and disadvantages of automation software packages for screening in systematic reviews. The outcomes show that the SRA-Endnote helper was the preferred software due to the fast response time, user-friendly set up, intuitive layout and the fact that it does not rely on internet connection. This study identified a number of relevant considerations for systematic review software packages, and individual systematic reviewers can take this into consideration whilst performing the citation screening and dispute resolution steps of a systematic review.

## Additional file

Additional file 1: **Table S1.** Qualitative questionnaire schedule. (DOCX 16 kb)

## Data Availability

All data generated or analysed during this study are included in this published article [and its supplementary information files].

## Abbreviation

SRA: Systematic Review Accelerator
