# Supplementary material for: Usability and acceptability of four systematic review automation software packages: a mixed method design
Source: Syst Rev. 2019 Jun 20;8:145. doi: 10.1186/s13643-019-1069-6 (PMC6587262; doi:10.1186/s13643-019-1069-6)
Supplement: Supplementary file 1 — Table S1. Qualitative questionnaire schedule. (DOCX 16 kb) [file 13643_2019_1069_MOESM1_ESM.docx]

Table S1 Qualitative questionnaire schedule

| **When screening citations for your systematic review, please consider:** | |
| --- | --- |
| 1 | How easy was it to learn how to use xxx?   1. Very difficult, 2. Difficult 3. Not so difficult 4. Easy 5. Very easy |
| 2 | What did you like about using xxx? |
| 3 | What did you dislike about using xxx? |
| 4 | What did you find easy about using xxx? |
| 5 | What did you find difficult about using xxx? |
| 6 | Were there any key features in xxx that you found particularly useful? |
| 7 | How would you rate the general usability of xxx?   1. Not at all user friendly 2. Not user friendly 3. Slightly user friendly 4. Fairly user friendly 5. Very user friendly |
| 8 | How would you rate the response time of xxx?   1. Very slow 2. Slow 3. Manageable 4. Quick 5. Very quick |
| 9 | Which software package did you find better for screening citations?   - Why did you make this selection |
| 10 | Which software package did you find better for resolving conflicts?   - Why did you make this selection |
| 11 | If you were to conduct a screening of citations again, which of the two software packages are you most likely to use?   - Why did you make this selection |
| 12 | Do you have any further comments or feedback? |
